# Microscopic and Crystallographic Analysis of Increased Acid Resistance of Melted Dental Enamel Using 445 nm Diode Laser: An Ex-Vivo Study

**DOI:** 10.3390/dj13080376

**Published:** 2025-08-19

**Authors:** Samir Nammour, Marwan El Mobadder, Aldo Brugnera, Praveen Arany, Mireille El Feghali, Paul Nahas, Alain Vanheusden

**Affiliations:** 1 Department of Dental Science, Faculty of Medicine, University of Liege, 4000 Liege, Belgium; 2 Laser Laboratory, Oral Surgery Department, Wroclaw Medical University, 50-425 Wroclaw, Poland; marwan.mobader@gmail.com; 3 Oral Biology, Surgery, and Biomedical Engineering, University at Buffalo, Buffalo, NY 14214, USA; prarany@buffalo.edu; 4 Department of Restorative and Esthetic Dentistry, Faculty of Dental Medicine, Lebanese University, Hadath Campus, Beirut 1003, Lebanonpaulnahas@yahoo.com (P.N.)

**Keywords:** caries prevention, caries resistance, enamel melting, preventive dentistry, diode laser, 445 nm, laser dentistry

## Abstract

**Background/Objectives**: This study aimed to evaluate the efficacy of a 445 nm diode laser in enhancing enamel resistance to acid-induced demineralization and to investigate the associated compositional and structural modifications using scanning electron microscopy (SEM), electron spectroscopy for chemical analysis (ESCA), and X-ray diffraction (XRD) crystallographic analysis. **Methods**: A total of 126 extracted human teeth were used. A total of 135 (*n* = 135) enamel discs (4 × 4 mm) from 90 teeth were assigned to either a laser-irradiated group or an untreated control group for SEM, ESCA, and XRD analyses. Additionally, 24 mono-rooted teeth were used to measure pulp temperature changes during laser application. Laser irradiation was performed using a 445 nm diode laser with a pulse width of 200 ms, a repetition rate of 1 Hz, power of 1.25 W, an energy density of 800 J/cm^2^, a power density of 3980 W/cm^2^, and a 200 µm activated fiber. Following acid etching, SEM was conducted to assess microstructural and ionic alterations. The ESCA was used to evaluate the Ca/P ratio, and XRD analyses were performed on enamel powders to determine changes in phase composition and crystal lattice parameters. **Results**: The laser protocol demonstrated thermal safety, with minimal pulp chamber temperature elevation (0.05667 ± 0.04131 °C). SEM showed that laser-treated enamel had a smoother surface morphology and reduced acid-induced erosion compared with controls. Results of the ESCA revealed no significant difference in the Ca/P ratio between groups. XRD confirmed the presence of hydroxyapatite structure in laser-treated enamel and detected an additional diffraction peak corresponding to a pyrophosphate phase, potentially enhancing acid resistance. Results of the spectral analysis showed the absence of α-TCP and β-TCP phases and a reduction in the carbonate content in the laser group. Furthermore, a significant decrease in the a-axis lattice parameter suggested lattice compaction in laser-treated enamel. **Conclusions**: Irradiation with a 445 nm diode laser effectively enhances enamel resistance to acid demineralization. This improvement may be attributed to chemical modifications, particularly pyrophosphate phase formation, and structural changes including prism-less enamel formation, surface fusion, and decreased permeability. These findings provide novel insights into the mechanisms of laser-induced enhancement of acid resistance in enamel.

## 1. Introduction

Dental caries prevention remains a major focus in restorative dentistry, with numerous protocols designed to enhance enamel and dentin resistance to acid attack. Traditional approaches primarily involve inducing morphological and structural changes on the enamel surface or promoting fluoride integration to strengthen the tooth structure and reduce caries risk [1,2]. In recent years, laser technology has emerged as a promising conservative method to improve the acid resistance of enamel. Specific laser wavelengths, applied with carefully controlled irradiation parameters, have been shown to induce beneficial changes in enamel, potentially increasing its resistance to demineralization caused by acids produced from bacterial biofilms on the tooth surface [3,4,5]. Such preventive laser treatments offer the advantage of being non-invasive while enhancing enamel resistance.

Previous studies have demonstrated that laser irradiation can induce chemical changes in the enamel phases and alter the enamel structure, suggesting that these modifications may enhance its acid resistance [6]. These changes may include surface melting, fusion, recrystallization, reduced solubility, and decreased carbonate phase within the hydroxyapatite crystals [6]. Despite these promising results, the exact mechanisms by which laser treatment confers enamel protection remain unclear [7]. Notably, the impact of laser irradiation on enamel’s fundamental structural unit (the prismatic structure, given by the hydroxyapatite crystal lattice) and the precise changes in its chemical composition have yet to be fully elucidated. In our discussion, we examined clinical and in vitro studies that have reported improved enamel caries resistance following treatment using various laser wavelengths (CO_2_, Nd:YAG, Er:YAG, Er,Cr:YSGG, Diode 980 nm, etc.). However, there remains a significant lack of comprehensive data on structural changes, as well as spectroscopic and crystallographic analyses, to clarify how enamel ultrastructure and phase composition may be beneficially altered [5,6,7,8,9]. This gap in the literature limits our understanding of the microscopic and chemical processes underlying laser-induced enamel acid resistance.

Furthermore, blue laser light has recently gained popularity in dental clinics and is widely available on the market at a relatively low cost. The low absorption of this wavelength by hydroxyapatite may help reduce structural alterations and the overall heat generated in irradiated enamel. Thus, there is a need to assess how other wavelengths improve the acid resistance of enamel with less eventual alteration in its structural and chemical properties.

Clarifying the mechanistic basis of acid resistance enhancement induced by blue laser light (445 nm) can support the development of novel, minimally invasive preventive strategies for managing dental caries with significant clinical relevance.

Therefore, in this study, we aimed to evaluate the effectiveness of a 445 nm diode laser in enhancing enamel acid resistance and to elucidate the underlying mechanisms responsible for this effect. Scanning electron microscopy (SEM) was used to assess morphological and structural enamel changes, while electron spectroscopy for chemical analysis (ESCA) was used to precisely determine the Ca/P ratio. X-ray diffraction (XRD) was used to investigate crystallographic modifications, including the formation of new phases and alterations in the enamel lattice. The null hypothesis states that no significant differences exist between the treated and untreated enamel samples.

## 2. Materials and Methods

### 2.1. Study Design

**Figure 1:**
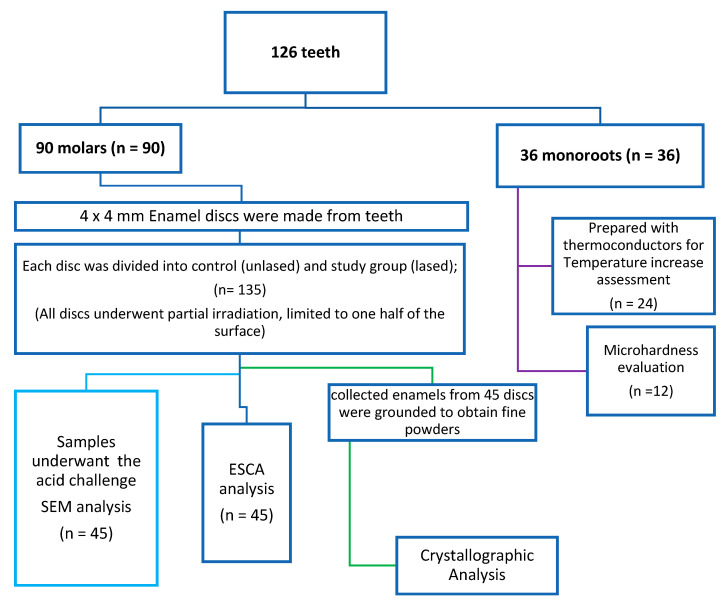
Illustration of the study design for SEM, ESCA, and crystallographic analysis.

This study investigated the effectiveness of a 450 nm diode laser in enhancing enamel resistance to acid attacks and aimed to characterize the chemical and structural changes induced by laser irradiation. A total of 126 extracted caries-free permanent human teeth were stored in a HEPES buffer solution (4 °C, pH 7.2) containing 2 mmol/L HEPES and 0.19 mmol/L sodium azide (NaN_3_). From 90 teeth, 135 enamel discs measuring 4 × 4 mm were prepared (Figure 1). Additionally, 24 single-root specimens were reserved for safety assessment, specifically to evaluate pulp temperature increase during laser exposure. Only 12 teeth were used to assess the microhardness of laser-treated and -untreated enamels. For scanning electron microscopy (SEM), electron spectroscopy for chemical analysis (ESCA), and the spectroscopy, 45 enamel discs were allocated to each technique (Figure 1).

**Figure 2:**
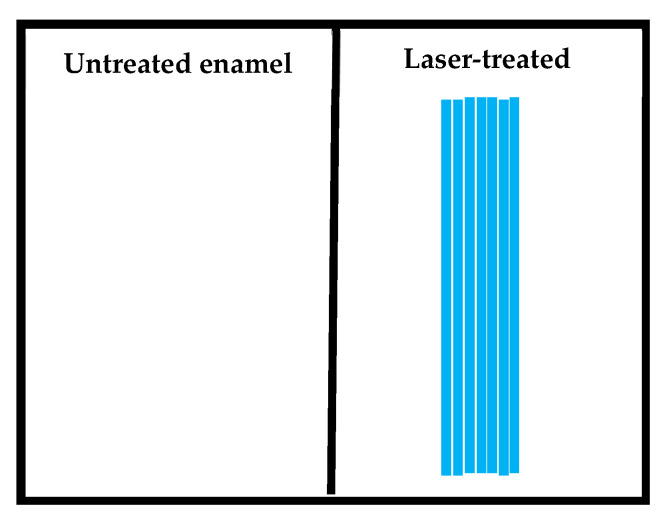
Illustration of 4 × 4 mm enamel disc samples (*n* = 135), showing both the control and study areas on the same sample. The blue lines represent a schematic view of the laser-irradiated area. A pattern was created on the enamel surface, every point of the pattern being formed by the impact of 1.25 W power, pulse duration of 200 ms, and a repetition rate of 1 Hz.

A fine-tip laboratory marking pen was used to delineate each enamel disc into two equal regions. One half of each disc was exposed to laser irradiation using predefined parameters, constituting the study group. The irradiation was applied in several parallel lines of 10 mm in length and 0.2 mm in width, confined to one side of the disc. This design allowed for direct comparison between laser-treated (study) and non-treated (control) surfaces on the same sample (Figure 2).

A total of 45 enamel discs were subjected to an acid etching challenge on both the laser-treated and non-treated regions using 37% phosphoric acid gel for 13 min. This procedure enabled surface morphological evaluation via scanning electron microscopy (SEM), allowing direct comparison between treated and untreated enamel areas on the same specimen.

In parallel, electron spectroscopy for chemical analysis (ESCA) was performed on a separate set of 45 non-etched discs (*n* = 45) to assess the chemical alterations induced by laser irradiation. Specifically, the calcium/phosphorus (Ca/P) ratio was calculated and compared between irradiated and non-irradiated regions (Figure 1).

An additional set of 45 non-etched discs, each with one half irradiated, was allocated for crystallographic analysis. Superficial layers of both lased and non-lased enamel areas were mechanically removed and finely ground into powders. These powders were analyzed to investigate potential changes in mineral phase composition and crystal lattice dimensions of hydroxyapatite resulting from laser treatment.

Furthermore, 24 extracted single-root teeth (*n* = 24) were used to evaluate pulp temperature increase during laser irradiation, under the same parameters used in the experimental protocol. Additionally, the other 12 extracted teeth (*n* = 12) were irradiated and used to evaluate the microhardness of the treated and untreated enamel. Informed consent was obtained from all patients, authorizing the use of their extracted teeth for research purposes. The collected teeth had been extracted for various reasons unrelated to our study and unknown to the authors. Since the research did not involve patients or animals, prior approval from the University Ethics Committee was not required.

### 2.2. Laser Irradiation

A diode laser with a wavelength of 445 nm (Yuwei Dental Laser, Yuwei Photoelectric Technology, Beijing, China) was employed for enamel irradiation. The delivered energy was verified using a calibrated laser power meter (Laser Power Meter, Gentec Electro-Optics, Quebec, QC, Canada). The irradiation was performed in contact mode using pulsed emission at a frequency of 1 Hz, with a pulse duration of 200 ms. The laser operated at an output power of 1.25 W, corresponding to an energy density of 800 J/cm^2^ and a power density of 3980 W/cm^2^. The optical fiber used had a diameter of 200 µm. Prior to each irradiation session, the fiber tip was activated using a red articulating paper. As previously described, laser application was restricted to a defined area on the enamel surface. The irradiation pattern consisted of multiple parallel lines of approximately uniform length, applied to the designated region (Figure 2).

### 2.3. Vickers Hardness Measurements

A total of 12 extracted human teeth (*n* = 12) with intact root structures were selected to assess and compare the hardness of laser-treated and -untreated enamel. A pyramidal diamond indenter with a square base, a divergence angle of 136°, and a load of 200 g was used.

### 2.4. Assessment of Protocol Safety

To assess the thermal safety of the laser protocol, 24 extracted human mono-root teeth with intact root structures and uncalcified root canals were selected (*n* = 24). The protocol was adapted from previously published methodologies [10], in which the apical portion of each root was removed, and the canals were instrumented to accommodate a 0.5 mm thermocouple probe, positioned at the ceiling of the pulp chamber in all samples. Prior to probe insertion, a thermoconductive paste (Warme Leitpaste WPN 10; Austerlitz Electronic, Nuremberg, Germany), with thermal conductivity comparable with that of soft tissues (0.1 cal·s^−1^·m^−1^·K^−1^), was introduced into the canal to ensure optimal thermal coupling between the probe and the surrounding dentin.

Probe positioning was radiographically verified to ensure reproducibility and accuracy. The root canal orifices were sealed with thermoplastic wax to maintain stable probe placement during laser irradiation. During irradiation, each root was immersed in a 37 °C water bath, with the cervical portion maintained above the waterline. The temperature stability of the water bath at 37 °C was continuously monitored and maintained.

Temperature measurements were obtained using a fast-response K-type thermocouple (HH806AWE, Omega, Manchester, UK) with an accuracy of ±0.1 °C. To validate probe placement reproducibility, one sample underwent four consecutive laser irradiations (5 s each), producing temperature rises of 37.1 °C, 37 °C, 37 °C, 37.1 °C, and 37 °C, confirming the consistency of the setup.

Subsequent thermal measurements were conducted using laser power settings ranging from 1 W to 4 W, with pulse durations of 0.2 s at 1 Hz, for a total irradiation time of 60 s. Temperature increases (Δ°TC) were calculated by subtracting the baseline temperature (37 °C) from the highest temperature recorded during irradiation. Mean values and standard deviations were computed. An intrapulpal temperature increase of ≤3 °C was considered within the threshold of biological safety for pulpal tissues.

### 2.5. Acid Etching Protocol

The enamel disc samples for acid application were etched using a 37% phosphoric acid gel, applied for a duration of 13 min to ensure adequate demineralization. Following the etching process, all samples (*n* = 45) were thoroughly rinsed with distilled water for 5 min to remove any residual acid and debris. This procedure aimed to create a uniform etching pattern, enhancing surface characteristics for subsequent analysis.

### 2.6. Analysis of Enamel Surface Morphology Using Scanning Electron Microscopy (SEM)

Following laser irradiation and subsequent acid challenge, a total of 45 enamel discs were subjected to morphological analysis to evaluate the effects of acid exposure on both laser-treated (study) and -untreated (control) enamel surfaces. Each disc included paired regions—lased and unlased—allowing for direct intra-sample comparison.

Surface characterization was performed using a SEM (JEOL 7600F, JEOL Ltd., Akishima, Japan) operated at an accelerating voltage of 15.0 kV. The objective was to assess the extent of acid-induced surface degradation and to detect any structural alterations attributable to laser irradiation. Representative samples from both groups were imaged at standardized magnifications. Surface features such as enamel prism integrity, porosity, surface smoothness, and signs of melting or recrystallization were documented and comparatively analyzed.

### 2.7. Electron Spectroscopy for Chemical Analysis (ESCA)

A total of 45 enamel discs, not subjected to acid exposure, were analyzed by ESCA to quantify the elemental composition of the enamel surface (*n* = 45). The measurements were performed using the VeraFlex^®^ III system (NO-VA, Bad Urach, Germany).

This analysis was conducted to determine the surface elemental composition of both laser-treated (study) and -untreated (control) areas. For each sample, twelve randomly selected points on the enamel surface were analyzed, and the mass percentages of detected elements were recorded, with particular focus on calcium and phosphorus, which are the principal constituents of enamel mineral content.

For each sample, twelve Ca/P atomic ratios were calculated for both the lased and unlased regions. Mean values and standard deviations were computed for comparative analysis between the two groups.

### 2.8. Crystallographic Analysis of Enamel Powders

For crystallographic analysis, enamel powders were obtained from a new set of 45 irradiated but non-etched teeth (distinct from those used for SEM analysis and ESCA). An enamel chisel was used to remove a superficial layer of non-irradiated and irradiated enamel by 3 successive scraps corresponding to 0.1–0.2 mm. The collected enamel pieces were ground into a fine powder of 100—50 µm (Pulverizer 4500G, Foshan Yajiarui Machinery Co., Foshan, China). This granule size was chosen to balance between sufficient diffraction intensity and preservation of the material’s native crystallinity. The powdered samples were analyzed using X-ray diffraction (XRD, Philips PW 1349/30, Amsterdam, Netherlands) to determine the crystalline phases present, primarily hydroxyapatite, and to assess changes in crystal structure and phase composition following laser irradiation. The diffractometer operated with Cu Kα radiation (λ = 1.5418 Å) and recorded diffraction patterns to evaluate the lattice parameters a and c, which characterize the 3D hexagonal geometry of the enamel apatite structure. Phase identification and lattice dimension analysis were based on Bragg’s law, expressed as follows:

*nλ* = 2*d* sin*θ*

where *n* is the diffraction order, *λ* is the wavelength of the incident X-ray beam, *d* is the interplanar spacing, and *θ* is the diffraction angle. This relationship enables precise calculation of crystal lattice dimensions and detection of any emerging or disappearing phases. The equation and methodology are based on established crystallographic principles as described by Cullity and Stock [11] and Klug and Alexander [12].

Bragg’s equation was used after the determination of interplanar spacings (*d_hkL_*) from the diffraction peaks to calculate the lattice parameters *a* and *c* in the hexagonal structure of hydroxyapatite. The general relation for a hexagonal lattice is as follows [12]:

$$\frac{1}{d_{hkl}^{2}}=\frac{4}{3}\cdot \frac{h^{2}+hk+k^{2}}{a^{2}}+\frac{l^{2}}{c^{2}}$$

where *d_hkL_* is the interplanar spacing calculated from the position of the diffraction peaks; *h*, *k*, and *L* are the Miller indices of the considered planes; and *a* and *c* are the lattice parameters to be determined.

Regarding the c-axis, its value can be independently determined from the a-axis parameter by analyzing the diffraction peaks at 002 and 310 positions. The estimation of the c-axis was further corroborated using the LaueScherrer equation, a widely employed method in X-ray diffraction and crystallography. This equation relates the crystallite size in a solid to the broadening of diffraction peaks, providing an additional means of verifying the c-axis measurement [13].

Crystal lattice parameters were determined, with particular attention to the dimensions of the a-axis and c-axis, which describe the hexagonal geometry of enamel lattice apatite. Determination of the c-axis required the analysis of two separate diffraction peaks (002 and 310 positions), allowing it to be distinguished from the a-axis.

### 2.9. Statistical Analysis

All data were analyzed using descriptive statistics to calculate mean values and standard deviations. A significant level of *p* < 0.05 was adopted for all statistical comparisons. For the evaluation of the pulpal temperature increase, mean Δ°TC values were compared across different laser power settings using repeated-measures ANOVA, followed by Tukey’s post hoc test to identify specific differences between conditions. For the ESCA, the Ca/P atomic ratios from lased and unlased regions were compared using the paired Student’s *t*-test to assess statistically significant differences in elemental composition. In the SEM analysis, surface morphology was qualitatively evaluated, and no inferential statistics were applied due to the descriptive nature of the data. For the crystallographic analysis, lattice parameters (a and c axes) were calculated from XRD data using Bragg’s law. Statistical comparison of values between groups was performed using the paired Student’s *t*-test to assess the significant differences. All analyses were performed using GraphPad prism 10 software to ensure rigor and reproducibility.

## 3. Results

### 3.1. Vickers Hardness Measurements

The average Vickers hardness value of the unlased enamels was 290 ± 45 kg/mm^2^, while that of laser-treated enamels was 525 ± 60 kg/mm^2^ (Table 1). The hardness of laser-treated enamel increased by approximately 50%. Results of the Student’s *t*-test showed a high significant difference between groups (Table 1).

### 3.2. Assessment of Protocol Safety

**Figure 3:**
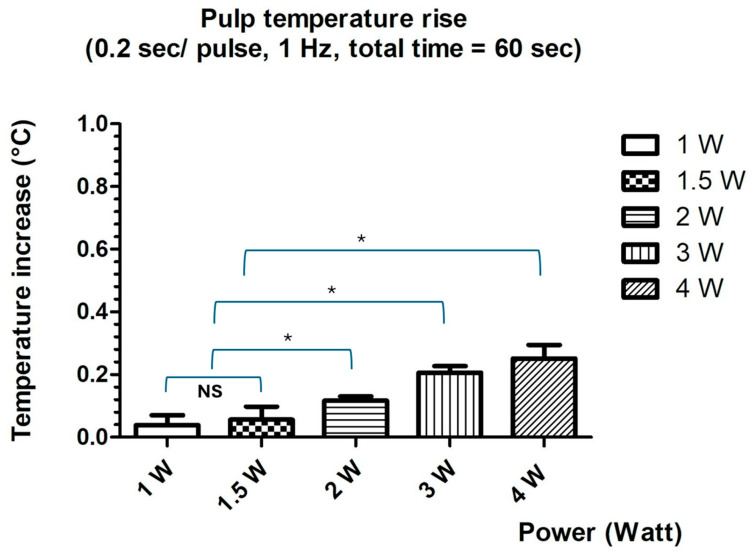
Mean values and standard deviations of temperature increase at pulp ceiling at different laser power settings. NS = No significant difference was observed; * (asterisk) = statistically significant differences were observed.

A gradual increase in mean temperature values was observed with increasing power settings, under consistent conditions (0.2 s per pulse, 1 Hz, total irradiation time: 60 s). At a power output of 1 W, the mean value was 0.038 ± 0.079 °C. This slightly increased to 0.057 ± 0.041 °C at 1.5 W. A more pronounced rise was noted at 2 W, with a mean value of 0.117 ± 0.014 °C, followed by further increases at 3 W (0.207 ± 0.021 °C) and 4 W (0.252 ± 0.043 °C). A repeated-measures ANOVA, followed by Tukey’s multiple comparisons post hoc test, was performed to assess statistical differences among the experimental groups. Results of the statistical analysis revealed significant differences among most groups (*p* < 0.05), except for the comparison between the 1 W and 1.5 W groups, where the difference was not statistically significant. Despite the observed increases, all measured values remained within a range considered safe for maintaining pulp vitality (Table 2, Figure 3).

### 3.3. Scanning Electron Microscopy (SEM)

**Figure 4:**
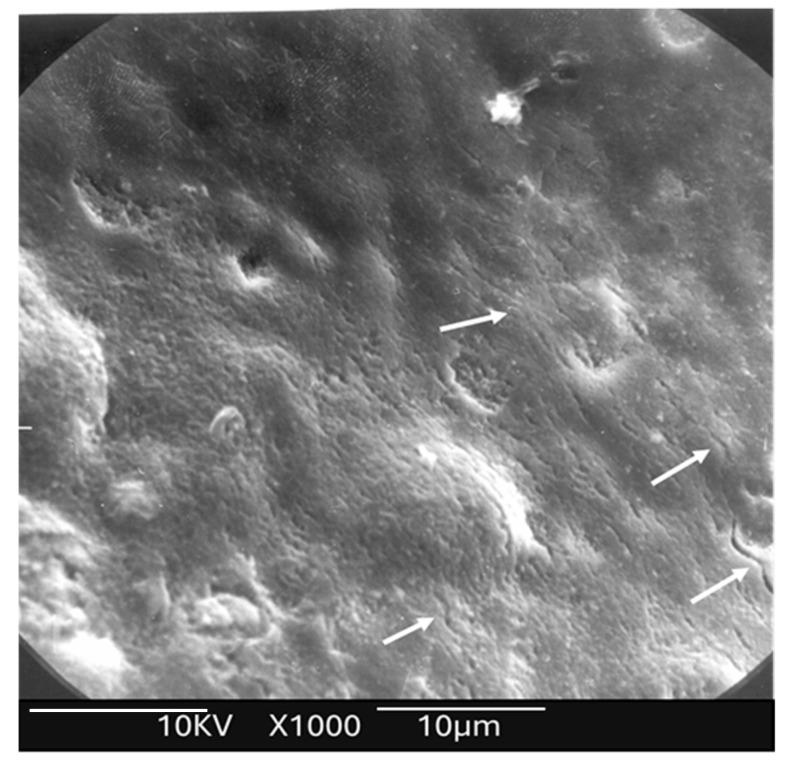
Scanning electron microscopy (SEM) analysis of the study group prior to acid application revealed evidence of enamel surface melting, characterized by the presence of multiple microcracks. White arrows show the existence of several cracks.

**Figure 5:**
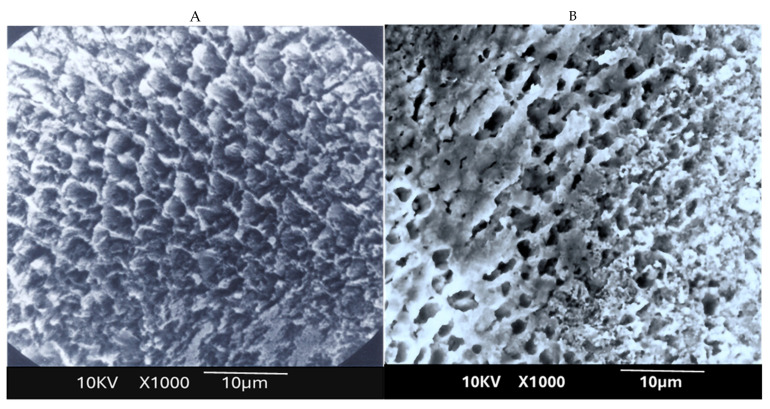
Scanning electron microscopy (SEM) analysis of the untreated enamel surface shows pronounced signs of acid-induced demineralization, with clear morphological alterations indicative of severe dissolution (**A**,**B**). These include loss of surface integrity, exposure of interprismatic spaces, and a distinctive “honeycomb” appearance. Open prism sheaths and tubule-like structures are evident, reflecting deep mineral loss and extensive enamel degradation.

**Figure 6:**
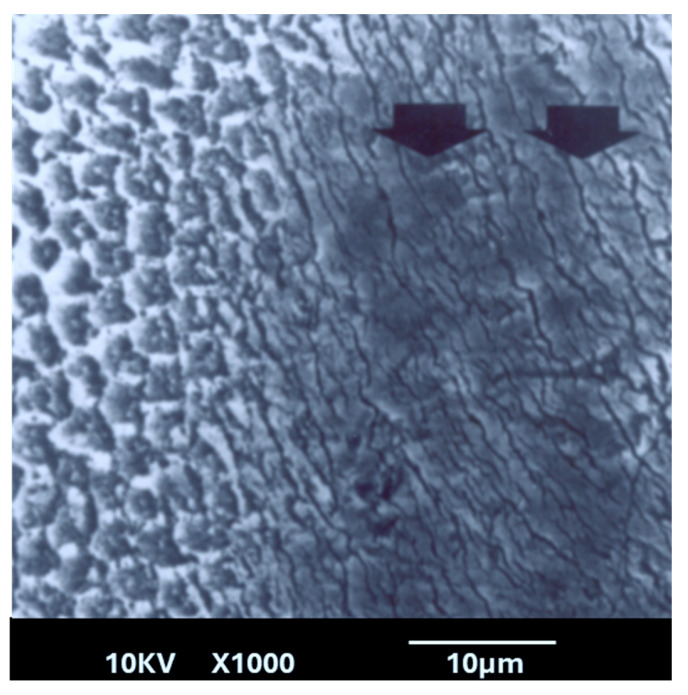
Scanning electron microscopy (SEM) image showing both the control group (left half) and the lased study group (right half) after acid application. Arrows indicate lased enamel that shows several cracks. The lased region appears comparatively less affected by acid-induced dissolution.

The lased enamel surface exhibited features consistent with melting, characterized by an irregular surface and the presence of multiple cracks (Figure 4). In the control group (unlased enamel), SEM examination revealed surface characteristics typical of enamel degradation caused by acid exposure (Figure 5). In contrast, the lased enamel in the study group showed a markedly reduced impact from the acid challenge (Figure 6). The enamel surface appeared smooth in most of the treated samples, suggesting that laser-induced melting remained stable even after acid exposure. It is also important to note that the severity of acid-induced etching varied among the samples, affecting both non-irradiated enamel and laser-melted areas (Figure 5 and Figure 6).

### 3.4. Electron Spectroscopy for Chemical Analysis (ESCA)

Results of the ESCA revealed the Ca/P integral ratios based on the peak areas corresponding to calcium and phosphorus in the obtained spectrum. The mean Ca/P ratio was 1.31 ± 0.013 for the control group, although a reduction in the ratio was observed in the lased group, resulting in a value of 1.14 ± 0.12. However, a paired Student’s *t*-test at a 95% confidence level indicated that the difference between the study (lased) and control (unlased) groups was not statistically significant (Table 3).

### 3.5. X-Ray Crystallographic Analysis

**Figure 7:**
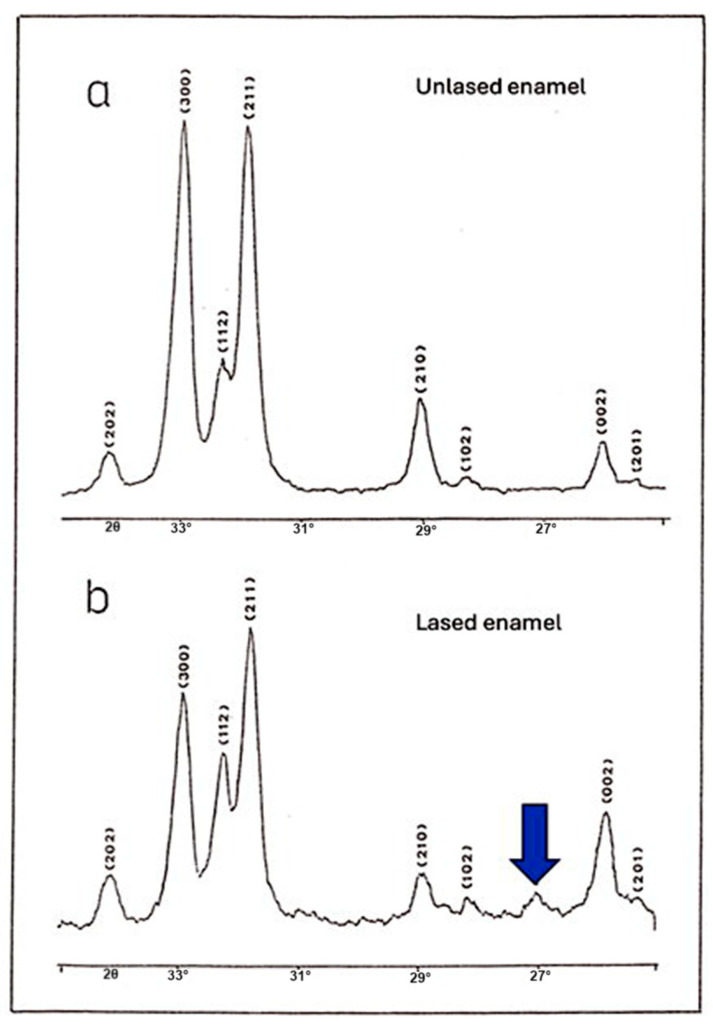
Diffraction spectrum (**a**) corresponds to unlased enamel. Spectrum (**b**), acquired from a second independent laboratory using lased enamel, confirms the presence of a new crystalline phase. This phase is characterized by a primary diffraction peak corresponding to α-Ca_2_P_2_O_7_ (α-phase calcium pyrophosphate), indicated by the blue arrow in spectrum (**b**).

The diffraction spectra (Figure 7) of both the control and study groups exhibited all characteristic peaks of hydroxyapatite, as described in the standard ASTM card No. 9-432. However, in the study group (lased), a new, additional small peak at 2θ = 27.05° was observed, which did not correspond to the hydroxyapatite found in the unlased enamel (Figure 7). This peak may be attributed to the maximum intensity peak of α-Ca_2_P_2_O_7_ (α-phase calcium pyrophosphate). Furthermore, the presence of the peak (which should be 70% of the intensity of the first peak in the absence of preferential orientation) cannot be confirmed, as it overlapped with the (203) peak of hydroxyapatite (cf. Index Inorganic to the Power Diffraction File, 1971).

The presence of this additional peak of calcium pyrophosphate in the diffraction spectrum of irradiated enamel powder was further corroborated by X-ray diffraction analysis performed at a second laboratory. Results of the spectral analysis revealed the absence of α-TCP (tricalcium phosphate) and β-TCP in the diffraction spectrum of the lased enamel and a reduction in the carbonate phase. The consistency of these values was confirmed through analysis conducted in two independent laboratories.

#### 3.5.1. A-Axis Parameter

The average value of the a-axis parameter for the study group (lased enamel) was 9.448 ± 0.00594 compared with 9.312 ± 0.00895 for the control group (unlased enamel). A paired Student’s *t*-test at a 95% confidence level revealed a statistically significant difference between the a-axis values of the lased and unlased enamel. This result indicates a contraction of the a-axis in the apatite crystal lattice following fusion and recrystallization induced by laser treatment (Table 4). Consequently, the lattice dimensions of the lased hydroxyapatite approached those of the stoichiometric hydroxyapatite.

#### 3.5.2. C-Axis Parameter

At a 95% confidence level, the mean values obtained for both the control and irradiated enamel groups did not significantly differ, as determined by a paired Student’s *t*-test. The mean c-axis parameter for the study group was 6.880 ± 0.0005 compared with 6.881 ± 0.002 for the control group. These results indicate that the c-axis of the apatite crystal lattice remained unaffected by laser irradiation of the enamel (Table 4).

**Table 4:** X-ray crystallographic analysis results. Different superscript letters indicate statistically significant difference (A, B, C…); similar superscript letters indicate the absence of a statistically significant difference (B; B…).

	Untreated Enamel (Control Group)		Laser-Treated Enamel (Study Group)	
Parameters	a-axis	c-axis	a-axis	c-axis
Mean value	9.448 ^A^	6.881 ^B^	9.312 ^C^	6.880 ^B^
Standard deviation	0.00594	0.00200	0.00895	0.00050

## 4. Discussion

Early childhood caries (ECC) is a significant global health problem, affecting nearly half of all preschool-aged children. The prevalence of ECC considerably varies between countries than between continents [14]. Results of a recent meta-analysis on the global prevalence of ECC estimated a random-effect pooled prevalence of 49% worldwide [15]. Chen et al. also demonstrated a wide variation in ECC prevalence across countries, confirming that ECC remains highly prevalent in most regions around the globe [16]. This persistently high prevalence has prompted researchers to explore and test new therapeutic approaches aimed at preventing or reducing the burden of dental caries in young children [16].

This study revealed several important new findings. The results of the scanning electron microscopy (SEM) analysis showed that enamel irradiated with a 445 nm diode laser exhibited significantly greater resistance to acid attack compared with non-irradiated enamel. In the control group (etched but non-treated enamel), the surface exhibited characteristics like those described in classic studies, such as that of Jongebloed et al. [17]; the worn enamel had lost all surface structure, with the presence of approximately hexagonal holes in the core of the crystallites, parallel to their long axis [17]. SEM images of the samples revealed that, in the control group, the inner part of the enamel prisms dissolved more rapidly than the outer part [17]. Furthermore, this dissolution was uneven, which can be explained at an even more microscopic level by the specific arrangement of the crystallites that constitute enamel. As for the study group, SEM images showed a melted enamel surface that was only slightly affected by acid etching. Thus, results of the SEM analysis confirm the effectiveness of laser irradiation in significantly increasing enamel resistance to acid challenges. These findings align with numerous other studies in the literature [18,19,20,21,22]. Moreover, it is important to note that the irradiation parameters used in this study do not cause a significant temperature rise in the dental pulp, with reported rises remaining below the critical threshold of 3 °C, thereby ensuring pulpal safety [10].

It can be argued that the most significant finding of this study came from the crystallographic analysis. Results of this analysis revealed a similarity between the diffraction spectra of irradiated and control enamel, with all identifiable peaks corresponding to hydroxyapatite, except for the presence of a new peak. This indicates that laser irradiation does not fundamentally alter the crystalline structure of enamel, at least in terms of the main phase composition. However, an additional small peak was detected in the spectrum of irradiated enamel. This peak is associated with the presence of pyrophosphate (α-Ca_2_P_2_O_7_), which forms at temperatures above 200 °C due to the condensation of hydrogen phosphate ions (HPO_4_^2−^) [23]. These phosphate ions are normally present in dental enamel at a proportion of approximately 4–5%, as reported in previous studies [23,24]. This finding is particularly relevant when considering the effects of laser irradiation on enamel solubility. The formation of pyrophosphate ions may be the primary factor enhancing enamel resistance to acid dissolution, which can explain the increased acid resistance observed in laser-irradiated samples [23,24]. Several hypotheses have been proposed to explain the increased acid resistance of enamel following laser irradiation. The interaction of laser light with enamel is a complex process involving photothermal and photochemical effects that depend on the laser’s wavelength, power, and irradiation parameters. At 445 nm, the diode laser energy is mainly absorbed by the organic matrix and minor chromophores within enamel and slightly absorbed by the enamel hydroxyapatite leading to localized heating. This heating can induce a melting and recrystallization of the enamel surface. Additionally, laser fiber activation produces dark chars around the fiber tip. During enamel irradiation, this char absorbs an important amount of the beam, causing a significant increase in the temperature around the tip.

This heat also contributes to the melting and recrystallization of the enamel surface. The fusion of enamel structures and the recrystallization of hydroxyapatite produce a new non-prismatic enamel arrangement. This process results in the disappearance of interprismatic spaces, which can delay the penetration of acid through its preferred pathways. In addition, the decomposition and dehydration of organic components, such as enamel proteins and water molecules, may reduce acid diffusion and delay its progression. Simultaneously, elevated temperatures facilitate phase transformations within the mineral component, including the reduction in carbonate phase and the condensation of hydrogen phosphate ions into pyrophosphate, as confirmed by the crystallographic findings in our study. This pyrophosphate formation enhances the enamel resistance to acid dissolution by inhibiting mineral breakdown. Furthermore, laser-induced thermal stress has led to the reorganization and compaction of the hydroxyapatite lattice, as evidenced by the observed contraction of the a-axis lattice parameter. This lattice compaction and the carbonate phase reduction may further increase enamel’s structural stability and reduce its solubility. Overall, the synergy of surface morphological fusion, chemical phase changes, and crystallographic lattice modifications underlies the increased acid resistance observed following 445 nm diode laser irradiation, providing a multifaceted defense against caries formation.

A classic study by Stern et al. [25] suggested that this effect results from decreased enamel permeability due to the fusion and to the disappearance of the preferred pathways for penetration and progression of acid through the enamel structure via the interprismatic and intercrystallite spaces. Similarly, Lenz et al. [26] reported findings that support this explanation. According to this hypothesis, laser irradiation enhances acid resistance by altering the enamel surface, without necessarily reducing its solubility. In a related theory, Yamamoto [27] proposed that the reduced permeability is due to the disintegration of the organic matrix around the prisms in fused enamel. Moreover, the elimination of water-containing organic enamel components during heating removes a potential pathway for hydrogen ion (H^+^) penetration. This idea is further supported by the findings of Sato et al. [28].

However, Borggreven et al. [29,30] challenged this perspective, demonstrating in multiple studies that melted enamel irradiated under similar conditions exhibited increased permeability. They argued that chemical transformations, rather than physical surface modifications, play a more significant role in enhancing acid resistance. Some evidence suggests that the formation of pyrophosphate (P_2_O_7_^4−^) is the key factor responsible for this increased resistance [29,30].

Pyrophosphate, which is formed through high-temperature-induced phase changes in enamel, significantly enhances enamel’s resistance to demineralization by altering its dissolution properties. It inhibits mineral breakdown and interferes with enamel dissolution mechanisms, which may explain the substantial reduction in acid susceptibility observed in our study [29,30]. This finding aligns with the work of Fowler [31] and other studies [32], who reported that heating enamel (in an oven) promotes the formation of pyrophosphate, thereby significantly improving enamel’s resistance to acid attack. These results underscore that those chemical changes, specifically the generation of pyrophosphate ions (P_2_O_7_^4−^), rather than permeability reduction, are the primary drivers of enhanced acid resistance in laser-irradiated enamel. Nevertheless, in addition to this chemical effect, morphological modifications also seem to play a role. Laser irradiation leads to surface fusion, reducing permeability by sealing natural pores and irregularities, thereby limiting acid penetration and ion diffusion, and creating an additional protective barrier against demineralization. Among these, the formation of pyrophosphate ions (P_2_O_7_^4−^) appears to be the most crucial factor. This chemical transformation is induced by high-temperature phase changes.

We observed a contraction of the a-axis in the apatite crystal lattice in the laser-irradiated dental enamel, with values significantly different from those of the control group. The shrinkage of the a-axis observed after laser irradiation can be hypothesized to result from multiple mechanisms. Firstly, the high temperatures generated during laser irradiation may lead to the disappearance of structural water molecules that are naturally incorporated within the enamel matrix. Structural water plays a role in maintaining the lattice parameters of hydroxyapatite crystals, and its removal can cause a slight contraction of the crystal lattice, notably along the a-axis. Secondly, another plausible hypothesis relates to the disappearance of primary dislocations within the enamel’s crystalline network. During natural enamel formation, the incorporation of carbonate ions into the hydroxyapatite lattice creates lattice imperfections and distortions. These imperfections contribute to slight expansions of the crystal parameters, including along the a-axis. Upon laser irradiation and subsequent rapid melting and resolidification, it is possible that these carbonate-associated dislocations diminish or are reorganized. This would favor the formation of a more stoichiometric and structurally ordered hydroxyapatite, with fewer crystal defects. Consequently, a more compact and less distorted crystal structure can develop, leading to the observed contraction of the a-axis. It is important to emphasize that these explanations remain hypothetical. Further in-depth structural and compositional analyses are required to definitively confirm these mechanisms.

As mentioned in the introduction, numerous studies have investigated the effects of laser irradiation with varying wavelengths and irradiation parameters on caries resistance. In this context, Cîrdei et al. [33] conducted a comparative in vitro study to evaluate the surface and mineral changes in primary enamel following diode laser irradiation and the application of remineralization agents. Their research focused on the effects of two different diode laser wavelengths, 808 nm and 980 nm, in conjunction with a pH cycling remineralization process. The findings revealed that the 808 nm diode laser exhibited greater efficacy in enhancing the weight percentage of calcium and phosphorus in enamel compared with the 980 nm diode laser [33]. This effect was particularly pronounced in the calcium weight percentage after the pH cycle remineralization treatment, suggesting that the 808 nm diode laser may be more effective in promoting enamel remineralization [31]. Moreover, Al-Omari et al. [34] observed that Nd: YAG laser irradiation resulted in a reduction in dentin microhardness values, even though the specimens were not submitted to the cariogenic challenge after irradiation [34]. Considering that the Nd: YAG laser irradiation by itself was unable to maintain hardness values like those found in the non-lased dentin, the subsequent cariogenic challenge performed in their study probably enhanced the decrease in microhardness, and, therefore, this laser showed less satisfactory results compared with the Er: YAG laser [34]. On the other hand, Feng et al. [29] published a Systematic Review and Meta-Analysis (SR-MA) on the effect of Er: YAG laser irradiation on preventing enamel caries. The SR-MA concluded that after Er: YAG laser irradiation, enamel can maintain higher surface microhardness in acidic environments, revealing its potential in preventing enamel caries [33]. However, based on the SR-MA, the effect of Er: YAG laser irradiation on the release of calcium ions in acidic solutions and the surface microhardness of demineralized enamel was not significant [29]. Here, it can be noted that the studies included in the SR-MA assessed the outcome by evaluating the surface microhardness, the calcium ions released, and the mineral loss by micro-CT [29]. Hence, although important, these included studies did not explain a clear mechanism of action for how the caries resistance is obtained.

Several studies have shown that enamel microhardness is a key indicator of its mineral integrity and mechanical strength, particularly in the context of acid resistance. An increase in microhardness is generally associated with a denser and less porous enamel structure, which limits acid progression and slows demineralization [29,35,36]. Treatments aimed at reinforcing the enamel microstructure, such as the application of remineralizing agents or physical methods like laser irradiation, have been shown to significantly enhance microhardness and, consequently, resistance to acid attacks [29,35,36].

We acknowledge that surface microhardness testing is an important parameter for evaluating mineral changes. Although our study demonstrated an increase in the microhardness of lased enamel, we did not assess the mineral content between groups to directly confirm the enhancement of acid resistance. Instead, we focused on the analysis of the morphological and chemical changes that may contribute to improved acid resistance. We conducted detailed microscopic, chemical, and crystallographic analyses to elucidate the structural and phase changes induced by 445 nm diode laser treatment. These investigations provide valuable insight into the mechanisms underlying enamel strengthening. Future research incorporating additional physical property assessments, such as fracture resistance testing, would offer complementary insights into the mechanical performance of laser-treated enamel under occlusal stress.

Our findings provide compelling evidence that may finally resolve the longstanding debate regarding the mechanism behind the increased acid resistance of laser-irradiated enamel, strongly suggesting that this enhancement results from the synergistic effect of both chemical and morphological changes. Specifically, the formation of pyrophosphate ions plays a fundamental role in stabilizing the enamel structure against acid attack, while morphological alterations, such as surface fusion and reduced permeability, reinforce this resistance by providing a physical barrier. Together, these mechanisms offer a more comprehensive explanation for the protective effect of laser irradiation on enamel. Future studies should focus on verifying the mechanical resistance and physical quality of laser-irradiated enamel by simulating oral conditions, particularly evaluating its susceptibility to fracture and its long-term persistence within the oral environment.

## 5. Conclusions

This study demonstrates that 445 nm diode laser irradiation significantly enhances enamel’s resistance to acid dissolution. Scanning electron microscopy revealed that laser-treated enamel exhibited a melted surface with minimal damage after acid exposure, unlike untreated enamel, which showed significant wear. Results of the crystallographic analysis indicated the presence of a new phase of pyrophosphate ions in irradiated enamel and a reduction in the carbonate phase. Our findings highlight those chemical changes but also a physical alteration producing a significant reduction in the a-axis size of the lased HAP. Our results support the potential of diode laser 445 nm irradiation in enhancing enamel resistance against acid challenges.

## Figures and Tables

**Table 1:** Mean values and standard deviations of Vickers hardness measurements for untreated and laser-treated enamel. Students’ *t*-test revealed a highly significant difference between the groups. Different superscript letters indicate statistically significant difference (A, B…); similar superscript letters indicate the absence of a statistically significant difference. Mean = mean value; SD = standard deviation.

Vickers Hardness	Untreated Enamel	Laser-Treated Enamel
Mean	290 ^A^	525 ^B^
SD	45	60

**Table 2:** Mean values and standard deviations at different laser power settings using 0.2 s per pulse, 1 Hz, and a total irradiation time of 60 s at different power settings.

Power (0.2 s/Pulse, 1 Hz Total Time: 60 s)	1 W	1.5 W	2 W	3 W	4 W
**Mean**	0.03833 ^A^	0.05667 ^A^	0.1167 ^B^	0.2067 ^C^	0.2517 ^D^
**Std. Deviation**	0.07935	0.04131	0.01366	0.02066	0.04262

Different superscript letters indicate statistically significant difference (A, B, C…); similar superscript letters indicate the absence of a statistically significant difference (A; or B; …). Mean = mean value; std. = standard deviation.

**Table 3:** Mean values and standard deviation of the Ca/P integral ratio values. Different superscript letters indicate statistically significant difference (A); similar superscript letters indicate the absence of a statistically significant difference (A; A).

	Mean Values	Standard Deviation
Control group	1.31 ^A^	0.0.13
Study group	1.14 ^A^	0.12

## Data Availability

The data is available on demand.

## References

[1] Dye B.A., Hsu K.L., Afful J. (2015). Prevalence and measurement of dental caries in young children. Pediatr. Dent..

[2] Dionysopoulos D., Koliniotou-Koumpia E., Tolidis K., Gerasimou P. (2017). Effect of fluoride treatments on bleached enamel microhardness and surface morphology. Oral Health Prev. Dent..

[3] Rodríguez-Vilchis L.E., Contreras-Bulnes R., Olea-Mejìa O.F., Sánchez-Flores I., Centeno-Pedraza C. (2011). Morphological and structural changes on human dental enamel after Er: YAG laser irradiation: AFM, SEM, and EDS evaluation. Photomed. Laser Surg..

[4] Steiner-Oliveira C., Rodrigues L.K., Soares L.E., Martin A.A., Zezell D.M., Nobre-Dos-Santos M. (2006). Chemical, morphological and thermal effects of 10.6-μm CO_2_ laser on the inhibition of enamel demineralization. Dent. Mater. J..

[5] Alkaisi A., Abdo S.B. (2021). Modification of Enamel Surface Morphology and Strength Using Nd:YAG Laser with Proper and Safe Parameters. Eur. J. Gen. Dent..

[6] Moshkelgosha V., Zandian R., Sohrabi M., Fekrazad R. (2024). Effect of CO_2_ Laser-Assisted Titanium Tetrafluoride on Demineralization of Enamel around Orthodontic Brackets. J. Lasers Med. Sci..

[7] Hamdi K., Elsebaai A., Abdelshafi M.A., Hamama H.H. (2024). Remineralization and anti-demineralization effect of orthodontic adhesives on enamel surrounding orthodontic brackets: A systematic review of in vitro studies. BMC Oral Health.

[8] Al-Maliky M.A., Frentzen M., Meister J. (2019). Artificial caries resistance in enamel after topical fluoride treatment and 445 nm laser irradiation. BioMed Res. Int..

[9] Pagano S., Lombardo G., Orso M., Abraha I., Capobianco B., Cianetti S. (2020). Lasers to prevent dental caries: A systematic review. BMJ Open.

[10] Namour A., El Mobadder M., Matamba P., Misoaga L., Magnin D., Arany P., Nammour S. (2024). The Safety of Removing Fractured Nickel-Titanium Files in Root Canals Using a Nd: YAP Laser. Biomedicines.

[11] Giliberti M., Lovisetti L. (2024). Wave Mechanics. Old Quantum Theory and Early Quantum Mechanics: A Historical Perspective Commented for the Inquiring Reader.

[12] Bragg W.L. (1949). The Crystalline State: A General Survey.

[13] Scherrer P. (1918). Bestimmung der Grosse und inneren Struktur von Kolloidteilchen mittels Rontgenstrahlen. Nach Ges. Wiss. Gottingen.

[14] Uribe S.E., Innes N., Maldupa I. (2021). The global prevalence of early childhood caries: A systematic review with meta-analysis using the WHO diagnostic criteria. Int. J. Paediatr. Dent..

[15] Maklennan A., Borg-Bartolo R., Wierichs R.J., Esteves-Oliveira M., Campus G. (2024). A systematic review and meta-analysis on early-childhood-caries global data. BMC Oral Health.

[16] Chen K.J., Gao S.S., Duangthip D., Lo E.C.M., Chu C.H. (2019). Prevalence of early childhood caries among 5-year-old children: A systematic review. J. Investig. Clin. Dent..

[17] Jongebloed W.L., Molenaar I., Arends J. (1975). Morphology and size-distribution of sound and acid-treated enamel crystallites. Calcif. Tissue Int..

[18] Badreddine A.H., Couitt S., Donovan J., Cantor-Balan R., Kerbage C., Rechmann P. (2021). Demineralization Inhibition by High-Speed Scanning of 9.3 µm CO_2_ Single Laser Pulses over Enamel. Lasers Surg. Med..

[19] Dias-Moraes M.C., Castro P.A., Pereira D.L., Ana P.A., Freitas A.Z., Zezell D.M. (2021). Assessment of the preventive effects of Nd: YAG laser associated with fluoride on enamel caries using optical coherence tomography and FTIR spectroscopy. PLoS ONE.

[20] Hassan M., Bakhurji E., AlSheikh R. (2021). Application of Er,Cr:YSGG laser versus photopolymerization after silver diamine fluoride in primary teeth. Sci. Rep..

[21] Li Z., Zhao J.Z., Li Q., Li C.L., Cai W., Chang J.L., Yang W.D. (2023). Effect of Neodymium-Doped Yttrium Aluminum Garnet Laser Combined with Desensitizing Toothpaste on Dentinal Tubule Occlusion Against Acid Challenge. Zhongguo Yi Xue Ke Xue Yuan Xue Bao..

[22] Yang N., Zhao Y. (2024). Study on the effect of crystal changes on acid resistance of erbium laser etched enamel surface. Dent. Mater. J..

[23] Foster B.L., Boyce A.M., Millán J.L., Kramer K., Ferreira C.R., Somerman M.J., Wright J.T. (2024). Inherited phosphate and pyrophosphate disorders: New insights and novel therapies changing the oral health landscape. J. Am. Dent. Assoc..

[24] Anastasiou A., Strafford S., Posada-Estefan O., Thomson C., Hussain S., Edwards T., Malinowski M., Hondow N., Metzger N., Brown C. (2017). β-pyrophosphate: A potential biomaterial for dental applications. Mater. Sci. Eng. C Mater. Biol. Appl..

[25] Stern R.H., Vahl J., Sognnaes R.F. (1972). Lased enamel: Ultrastructural observations of pulsed carbon dioxide laser effects. J. Dent. Res..

[26] Lenz P., Gilde H., Walz R. (1982). Untersuchungen zur Schmelzversiegelung mit dem CO_2_-Laser [Enamel sealing studies with the CO_2_ laser]. Dtsch. Zahnarztl. Z..

[27] Yamamoto H., Sato K. (1980). Prevention of dental caries by acousto-optically Q-switched Nd: YAG laser irradiation. J. Dent. Res..

[28] Sato K. (1979). Study of an asymmetric ESR signal in x-irradiated human tooth enamel. Calcif. Tissue Int..

[29] Feng Z., Yuan R., Cheng L., Fan H., Si M., Hao Z. (2024). Effect of Er:YAG Laser Irradiation on Preventing Enamel Caries: A Systematic Review and Meta-Analysis. Int. Dent. J..

[30] Borggreven J.M., Driessens F.C., van Dijk J.W. (1980). Diffusion through bovine tooth enamel as related to the water structure in its pores. Arch. Oral Biol..

[31] Fowler M.L., Ingram-Smith C., Smith K.S. (2012). Novel Pyrophosphate-Forming Acetate Kinase from the Protist *Entamoeba histolytica*. Eukaryot. Cell.

[32] Tareq M.S., Hamad T.K. (2024). In Vitro Studies on the Influence of Nd:YAG Laser on Dental Enamels. Lasers Med. Sci..

[33] Cîrdei M.V., Margan M.M., Margan R., Ban-Cucerzan A., Petre I., Hulka I., Horhat R.M., Todea D.C. (2024). Surface and Mineral Changes of Primary Enamel after Laser Diode Irradiation and Application of Remineralization Agents: A Comparative In Vitro Study. Children.

[34] Al-Omari W.M., Palamara J.E. (2013). The effect of Nd:YAG and Er,Cr:YSGG lasers on the microhardness of human dentin. Lasers Med. Sci..

[35] Akkus A., Karasik D., Roperto R. (2017). Correlation between micro-hardness and mineral content in healthy human enamel. J. Clin. Exp. Dent..

[36] Mansoor A., Moeen F., Mehmood M., Ul Hassan S.M., Ullah M.U., Khan T. (2019). Correlation between micro-hardness and mineral content in the healthy tooth enamel of humans belonging to different age groups. Pak. Armed Forces Med. J..

